# Colon Cancer-Related Genes Identification and Function Study Based on Single-Cell Multi-Omics Integration

**DOI:** 10.3389/fcell.2021.789587

**Published:** 2021-11-25

**Authors:** Xuepu Sun, Yu Guo, Yu Zhang, Peng Zhao, Zhaoqing Wang, Zheng Wei, Haiquan Qiao

**Affiliations:** ^1^ The First Affiliated Hospital of Harbin Medical University, Harbin, China; ^2^ School of Computer Science and Technology, Harbin Institute of Technology, Harbin, China; ^3^ Department of Neurosurgery, General Hospital of Heilongjiang Province Land Reclamation Bureau, Harbin, China; ^4^ School of Life Science and Technology, Harbin Institute of Technology, Harbin, China

**Keywords:** colon cancer, single cell, transcriptome, methylation, comprehensive analysis, marker genes

## Abstract

Transcriptomes and DNA methylation of colon cancer at the single-cell level are used to identify marker genes and improve diagnoses and therapies. Seven colon cancer subtypes are recognized based on the single-cell RNA sequence, and the differentially expressed genes regulated by dysregulated methylation are identified as marker genes for different types of colon cancer. Compared with normal colon cells, marker genes of different types show very obvious specificity, especially upregulated genes in tumors. Functional enrichment analysis for marker genes indicates a possible relation between colon cancer and nervous system disease, moreover, the weak immune system is verified in colon cancer. The heightened expression of markers and the reduction of methylation in colon cancer promote tumor development in an extensive mechanism so that there is no biological process that can be enriched in different types.

## Introduction

Colorectal cancer is the most common gastrointestinal tumor with high appearance, recurrence, and death. Up to 70% occurs in the colon, and according to the statistics of 36 cancers in 185 countries in 2018, colorectal cancer resulted in 881,000 deaths, of which over 550,000 cases were due to colon cancer. In addition, more than 1,000,000 new colon cancer patients were confirmed during that year, justifying why the screening and treatment of colon cancer are hot issues worldwide (Bailey et al., 2015; Chen et al., 2016; Bray et al., 2018; Fang et al., 2019; Liang et al., 2019; Liu et al., 2020; Hu et al., 2021a). With the development of different kinds of treatment technologies, the 5-year survival rate of early-stage colon cancer patients has increased up to 90%, however, many urgent problems remain to be solved: for example, the median survival time of colon cancer patients with metastasis is less than 2 years, and 5-year overall survival rate is only 50–65%. Early screening and diagnosis for colon cancer mainly depend on electronic colorectal mirror surgery and pathology, which is expensive and has poor prognosis (Kiriyama et al., 2012; Chiba et al., 2014; Missiaglia et al., 2014; Fakih, 2015; Miller et al., 2016; Sewda et al., 2016; Marmol et al., 2017; White et al., 2017; Zhao et al., 2017; Hu et al., 2020; Mo et al., 2020; Hu et al., 2021a; Hu et al., 2021b). As a result, molecular biomarkers are vital for verifying the mechanism that promotes colon cancer development and improving early screening, diagnosis, and treatment for colon cancer.

More and more research has revealed that abnormal epigenetic modifications contribute to the occurrence and development of colon cancer by changing the expression of genes associated with tumors. This has meant that the stable and critical epigenetic modification style, methylation, has become most viable candidate for colon cancer biomarker research (Cheng et al., 2018; Hong, 2018; Tang et al., 2018; Wang et al., 2019). The abnormal DNA methylation pattern associated with colon cancer has been verified (Simmer et al., 2012; Ao et al., 2019; Zuo et al., 2020). High and low methylated CpG sites have been identified using the data of Illumina Human Methylation 450K (Naumov et al., 2013). The Wnt signaling pathway (Novellasdemunt et al., 2015; Sheng et al., 2021), Rap1 signaling (Zhang et al., 2017), and many other signaling pathways have been confirmed to have an effect on colon cancer, at the same time, *GDNF* (Zhong et al., 2018), *BMP3* (Park et al., 2016), and a few other genes have been regarded as the signs of malignant colon cancer, while *NEU1* (Uemura et al., 2009) as well as others suppress metastasis.

A few genes have been recognized as the marker genes for colon cancer, however, the number of marker genes is not enough for choosing efficient target genes, and tumor heterogeneity has had an influence on the result. Considering the reasons mentioned above, in this study, we attempt to identify marker genes for colon cancer by the comprehensive analysis of transcriptomes and methylation at the single-cell level, and conduct functional enrichment analysis for the makers.

## Methods

### Dataset

The data used in this research are from a previous study (Bian et al., 2018) and are the Supplementary Material of the accession number GSE97693 in the Gene Expression Omnibus (GEO) database (Barrett et al., 2005) (https://www.ncbi.nlm.nih.gov/geo/).

### Data Preprocessing

Colon cancer cells which possess transcriptomes with methyl-omics and normal cells are selected for further analysis. The genes expressed in all cells are reserved in the analysis for RNA, in contrast, because of the poor quality of methylation sequencing, the genes methylated in over 50% of normal cells and cancer cells are reserved for further methylation analysis. The downloaded transcriptomes are normalized to FPKM or TPM, so the data are renormalized, and the expression level of each gene is represented uniformly by the expression level of each gene taking up the percentage of the average expression level of all genes in the cell. The DNA methylation level is represented by the average methylation level of CpG loci in the promoter region.

### Differentially Expressed Genes

Differentially expressed genes are defined by *p*-value <0.01 [Wilcox test (Hiller, 1988)] and |log2(FC)|>2 in this study. This experiment includes two parts: comparison between normal cells and tumor cells, and comparison between normal cells and different types of colon cancer cells. According to RNA, colon cancer cells are clustered into subtypes by Seurat (Butler et al., 2018) (version 3.0.1), which is a package in R specially designed for single-cell transcriptomic data across different conditions, technologies, and species.

### Differentially Methylated Genes

In this study, the differentially methylated genes are defined by the *p*-value threshold of the Wilcox test (Hiller, 1988) which is 0.01 and the absolute value of difference threshold which is 0.2. This module includes two comparisons: normal cells versus colon cancer cells and normal colon cells versus different types of cancer cells. The subtypes are consistent with the result of transcriptomic data clusters by default.

### Differentially Expressed and Methylated Genes

According to previous studies, DNA methylation is an important factor of changing the expression of genes. Gene expression is usually negatively regulated by the DNA methylation level. So the methylation and transcriptome genes with negative correlations are recognized as the marker genes for colon cancer as well as subtypes.

### Functional Enrichment Analysis

Functional enrichment analysis is performed for the marker genes to study their mechanism of affecting colon cancer development via the package in R called ClusterProfiler (version 3.12.0) (Yu et al., 2012). The R package ClusterProfiler is designed for comparing biological themes among genes clusters, such as those in Gene Ontology (GO), Kyoto Encyclopedia of Genes and Genomes (KEGG), and DO.

## Results

### Subtypes of Colon Cancer Cells

**FIGURE 1:**
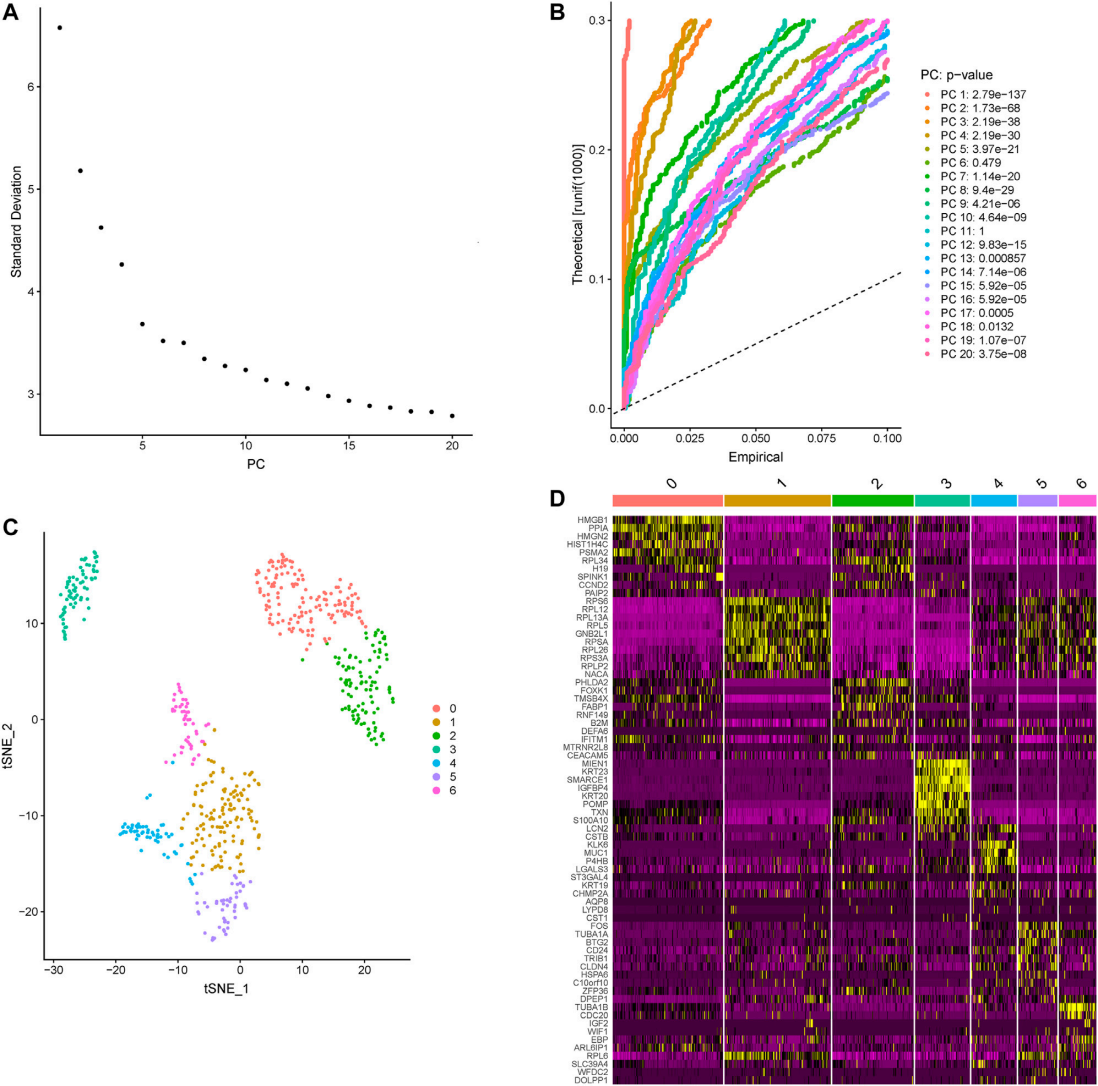
The subtypes clustered across all colon cancer cells. **(A)** The standard deviations of the top 20 principle components are shown, and the elbow corresponds well with the significant dims. **(B)** Statistical significance of the PCA scores of the top 20 PCs, the *p*-values are shown on the right. **(C)** The clusters of colon cancer are marked by color. **(D)** The heatmap of the top 10 differentially expressed genes in each subtype, yellow and purple represent high and low expression separately, the subtypes are marked by number.

Based on the Seurat R package, principle component analysis (PCA) of colon cancer cell transcriptomes is performed and the top five principle components are picked for later t-distributed stochastic neighbor embedding (t-SNE) (Figures 1A,B). A total of 607 colon cancer cells are clustered into 7 subtypes (Figure 1C). Contrasting with all other subtypes, differentially expressed genes in each subtype are identified and the top 10 cluster the colon cancer cells very well (Figure 1D; Table 1).

**TABLE 1:** The top 10 differentially expressed genes in each subtype.

Cluster 0	Cluster 1	Cluster 2	Cluster 3	Cluster 4	Cluster 5	Cluster 6
HMGB1	RPS6	PHLDA2	MIEN1	KLK6	FOS	TUBA1B
PPIA	RPL12	FOXK1	KRT23	MUC1	TUBA1A	CDC20
HMGN2	RPL13A	TMSB4X	SMARCE1	P4HB	BTG2	IGF2
HIST1H4C	RPL5	FABP1	IGFBP4	LGALS3	CD24	WIF1
PSMA2	GNB2L1	RNF149	KRT20	ST3GAL4	TRIB1	EBP
RPL34	RPSA	B2M	POMP	KRT19	CLDN4	ARL6IP1
H19	RPL26	DEFA6	TXN	CHMP2A	HSPA6	RPL6
SPINK1	RPS3A	IFITM1	S100A10	AQP8	C10orf10	SLC39A4
CCND2	RPLP2	MTRNR2L8	LCN2	LYPD8	ZFP36	WFDC2
PAIP2	NACA	CEACAM5	CSTB	CST1	DPEP1	DOLPP1

### Study for Differentially Expressed Genes

**FIGURE 2:**
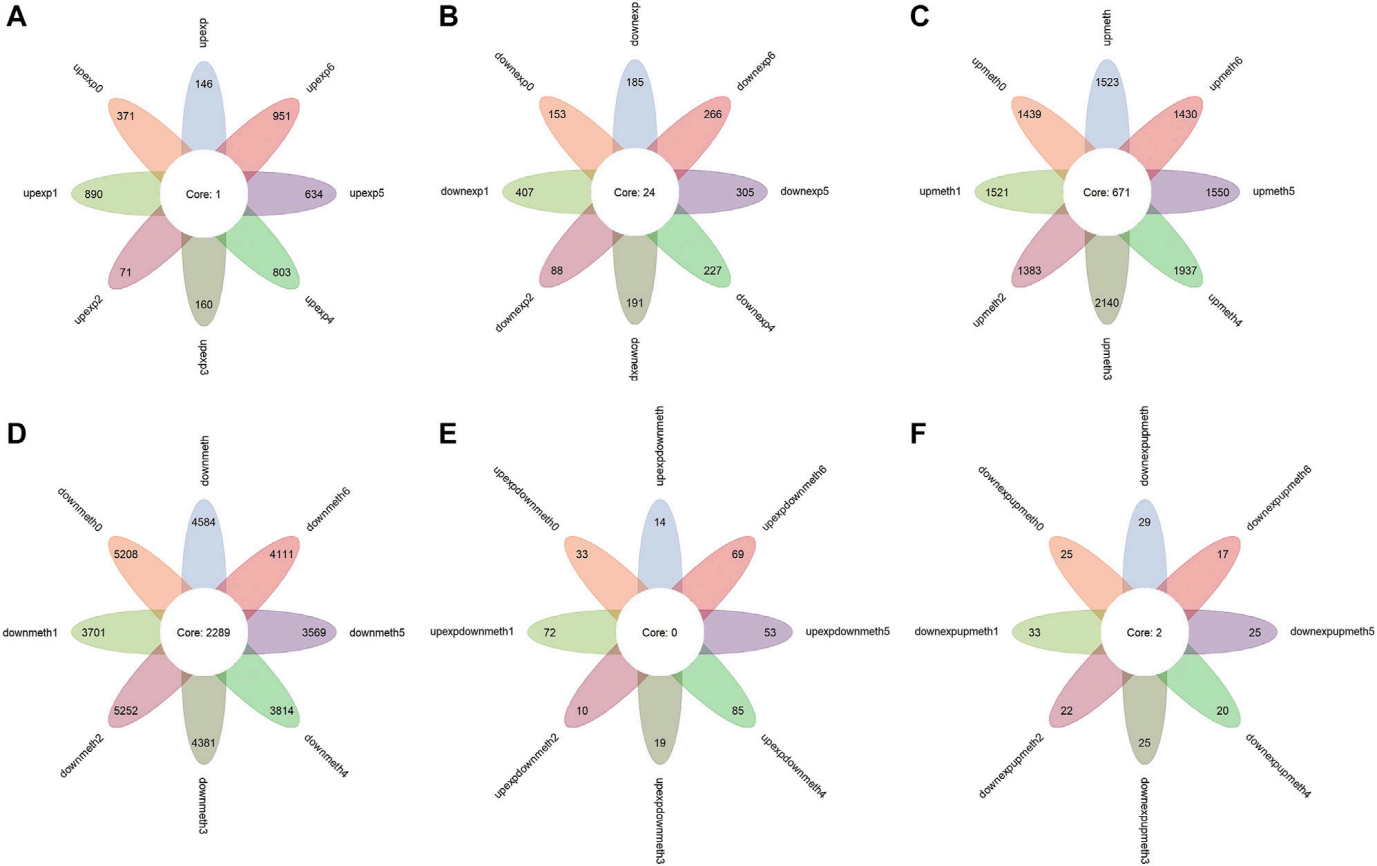
Functional enrichment analysis for dysregulated genes. The color of the point means the significance (*p*-value). The size of the point means the gene number. **(A)** The biological process enrichment of upregulated expressed genes in colon cancer and subtypes. **(B)** The biological process enrichment of downregulated expressed genes in colon cancer and subtypes. **(C)** The KEGG pathway enrichment of downregulated expressed genes in colon cancer and subtypes. **(D)** The biological process enrichment of upregulated methylated genes in colon cancer and subtypes. **(E)** The biological process enrichment of downregulated methylated genes in colon cancer and subtypes. **(F)** The KEGG pathway of downregulated methylated genes in colon cancer and subtypes. The biological process enrichment of upregulated expressed and downregulated methylated genes in colon cancer and subtypes. The biological process enrichment of downregulated expressed and upregulated methylated genes in colon cancer and subtypes.

The Wilcox test and fold change test are carried out between the 607 colon cancer cells and 20 normal cells with transcriptomes. In total, 331 differentially expressed genes are identified, 146 of which are upregulated in colon cancer (Figures 2A,B). To remove the influence of tumor heterogeneity, comparisons between normal cells and each subtype are performed. The result shows there are more differently expressed genes in each subtype in general, especially the genes of upregulated expression in tumors (Figures 2A,B), furthermore, variation in the amount of upregulated genes in subtypes is significant, this suggests that the genes whose expression is upregulated in tumors are significantly affected by subtypes.

At the same time, functional enrichment analysis indicates the genes of upregulated expression in colon cancer cells are enriched in a variety of biological processes, besides this, these processes are very different between subtypes. The findings above verify the diversities between subtypes and the different mechanisms of colon cancer development in subtypes (Supplementary Figure S1; Table 2). However, the genes of downregulated expression in colon cancer are enriched in fewer processes and for some subtypes, the enriched biological processes are extremely similar (Supplementary Figure S2; Table 2). The notable result in the KEGG function analysis shows that a few downregulated expression genes in some subtypes are enriched in Alzheimer disease, Huntington disease, and Parkinson disease, which reminds us of the possible relationship between the colon and nervous system disease (Supplementary Figure S3; Table 2).

**TABLE 2:** The enriched term number of function analysis for dysregulated genes.

		Colon cancer	Cluster 0	Cluster 1	Cluster 2	Cluster 3	Cluster 4	Cluster 5	Cluster 6
Biological process	Upexp	3	209	86	42	36	156	0	321
	Downexp	13	13	151	24	11	110	161	143
	upmeth	27	32	1	1	157	139	0	8
	downmeth	17	27	11	32	26	26	10	14
	upexpdownmeth	1	8	0	0	7	0	1	1
	downexpupmeth	25	9	40	39	3	0	73	34
KEGG	downexp	4	5	34	2	5	21	20	15
	downmeth	4	9	1	9	9	6	1	3

### Study of Differentially Methylated Genes

The *p*-value of the Wilcox test and the absolute value of difference between normal cells and colon cancer cells are verified. Overall, 1,523 genes of upregulated methylation and 4,584 genes of downregulated methylation are identified (Figures 2C,D). Comparing normal colon cells and colon cancer cells of different types, the result shows that a similar number of differentially methylated genes are recognized in subtypes (Figures 2C,D), which suggests the pattern of dysregulated methylation in colon cancer is similar.

In addition, the biological process functional enrichment shows that the genes of upregulated methylation in colon cancer mainly act on two fields: first, influencing different signaling transmission using different means, such as the neuroactive ligand−receptor interaction, calcium signaling pathway, pathways verified to be related to colon cancer, for example, the cAMP signaling pathway (Leiphrakpam et al., 2018), and cell adhesion molecules (CAMs) (Hara et al., 2019); second, cell−cell adhesion *via* plasma−membrane adhesion molecules, which is enriched in almost all subtypes. Another important finding is that some marker genes have an effect on cognition, learning or memory, and regulation of the neurological system process, which also suggests that colon cancer may be related to nervous system disease, such as Alzheimer disease and Parkinson disease (Supplementary Figure S4). In both biological process and KEGG pathways, the functions of downregulated methylation genes in tumors are highly consistent, including the perception of smell, cytokine−cytokine receptor interaction, and a few others (Supplementary Figures S5, 6).

### Study of Marker Genes

In light of existing studies, abnormal methylation change leading to dysregulated expression of genes is a vital early event in the development of certain kinds of cancers, and, in general, RNA and methylation have a negative relationship. Therefore, 14 genes with upregulated expression together with downregulated methylation and 29 genes with downregulated expression as well as upregulated methylation are identified as marker genes for colon cancer (Figures 2E,F). The number of upregulated expression and downregulated methylation genes in subtypes has a large fluctuation, but the number of downregulated expression and upregulated methylation genes in subtypes is almost same (Figures 2E,F), which is consistent with the result of differentially expressed genes. In addition, this indicates that heterogeneity in colon cancer is more reflected in genes whose expression is upregulated.

In function analysis, upregulated expression and downregulated methylation genes promote colon cancer in extensive mechanisms so that there is no term enriched in some subtypes, moreover, the mechanisms are different in subtypes (Supplementary Figure S7). The functions of downregulated expression and upregulated methylation genes are mainly associated with immunity, such as innate immune response−activating signal transduction, activation of innate immune response, neutrophil activation involved in immune response, and negative regulation of interleukin−1 secretion (Supplementary Figure S8), which is in line with our study.

## Discussion

According to existing studies, some genes and pathways have been confirmed to be related to colon cancer. However, those studies are mostly dependent on the data of bulk sample sequencing. Therefore, in this study, the single-cell transcriptomes and methylation of colon cancer are used to remove the influence of tumor heterogeneity. Differentially expressed and methylated genes are identified as marker genes for subtypes. Biological process analysis identifies several processes, which suggest the relationship between colon cancer and diseases of the nervous system (Alzheimer disease, Parkinson disease). In addition, the intra-tumor heterogeneity of colon cancer is more reflected in upregulated expressed genes. Clinically, this finding provides possible targeted therapeutic approaches for colon cancer patients. However, the small number of cells and the high intra-tumor heterogeneity mean that the findings need to be verified in larger cohorts.

## Data Availability

The datasets presented in this study can be found in online repositories. The names of the repository/repositories and accession number(s) can be found below: https://www.ncbi.nlm.nih.gov/geo/, GSE97693.

## Supplementary Material

Supplementary Figure S1 The results of biological process enrichment analysis for upregulated expressed genes in colon cancer and subtypes.

Supplementary Figure S2 The results of biological process enrichment analysis for downregulated expressed genes in colon cancer and subtypes.

Supplementary Figure S3 The results of KEGG pathway enrichment analysis for downregulated expressed genes in colon cancer and subtypes.

Supplementary Figure S4 The results of biological process enrichment analysis for upregulated methylated genes in colon cancer and subtypes.

Supplementary Figure S5 The results of biological process enrichment analysis for downregulated methylated genes in colon cancer and subtypes.

Supplementary Figure S6 The results of KEGG pathway enrichment analysis for upregulated methylated genes in colon cancer and subtypes.

Supplementary Figure S7 The results of biological process enrichment analysis for upregulated expressed and downregulated methylate genes in colon cancer and subtypes.

Supplementary Figure S8 The results of biological process enrichment analysis for downregulated expressed and upregulated methylated genes in colon cancer and subtypes.
