# Supplementary figures and images for: Colon Cancer-Related Genes Identification and Function Study Based on Single-Cell Multi-Omics Integration
Source: Front Cell Dev Biol. 2021 Nov 25;9:789587. doi: 10.3389/fcell.2021.789587 (PMC8657154; doi:10.3389/fcell.2021.789587)

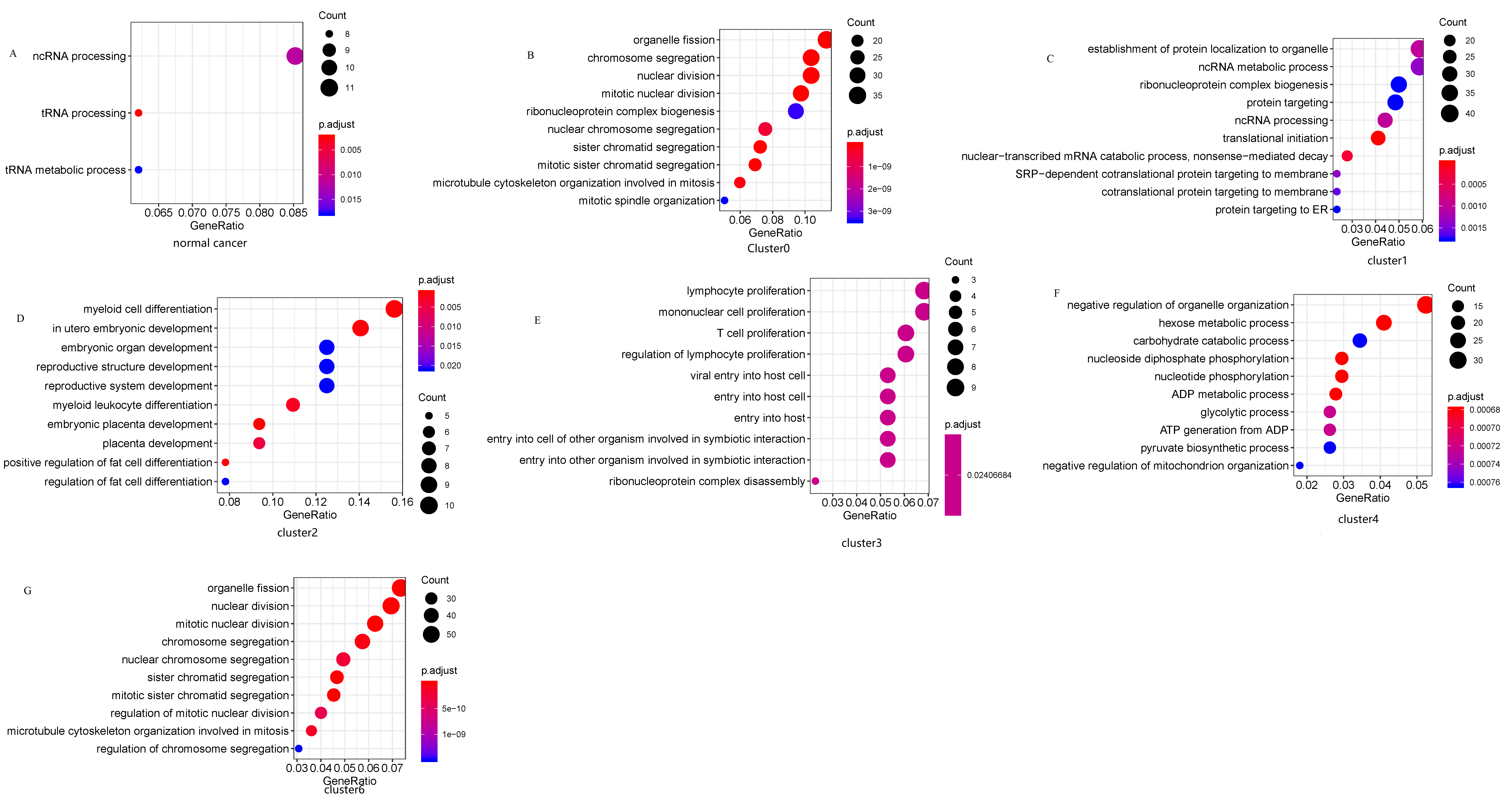

Supplement: Supplementary file 1 [file Image1.TIFF]

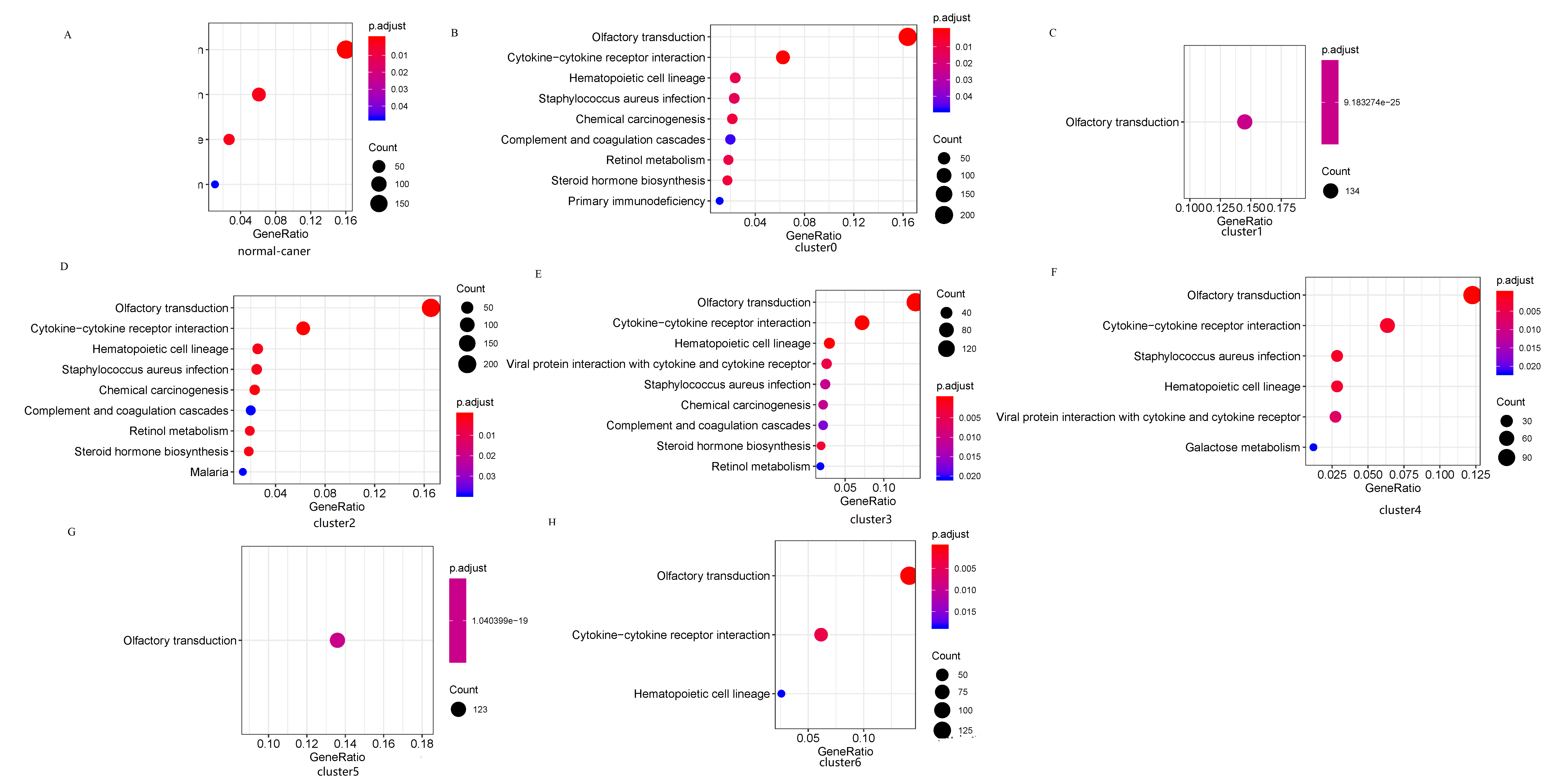

Supplement: Supplementary file 2 [file Image6.TIF]

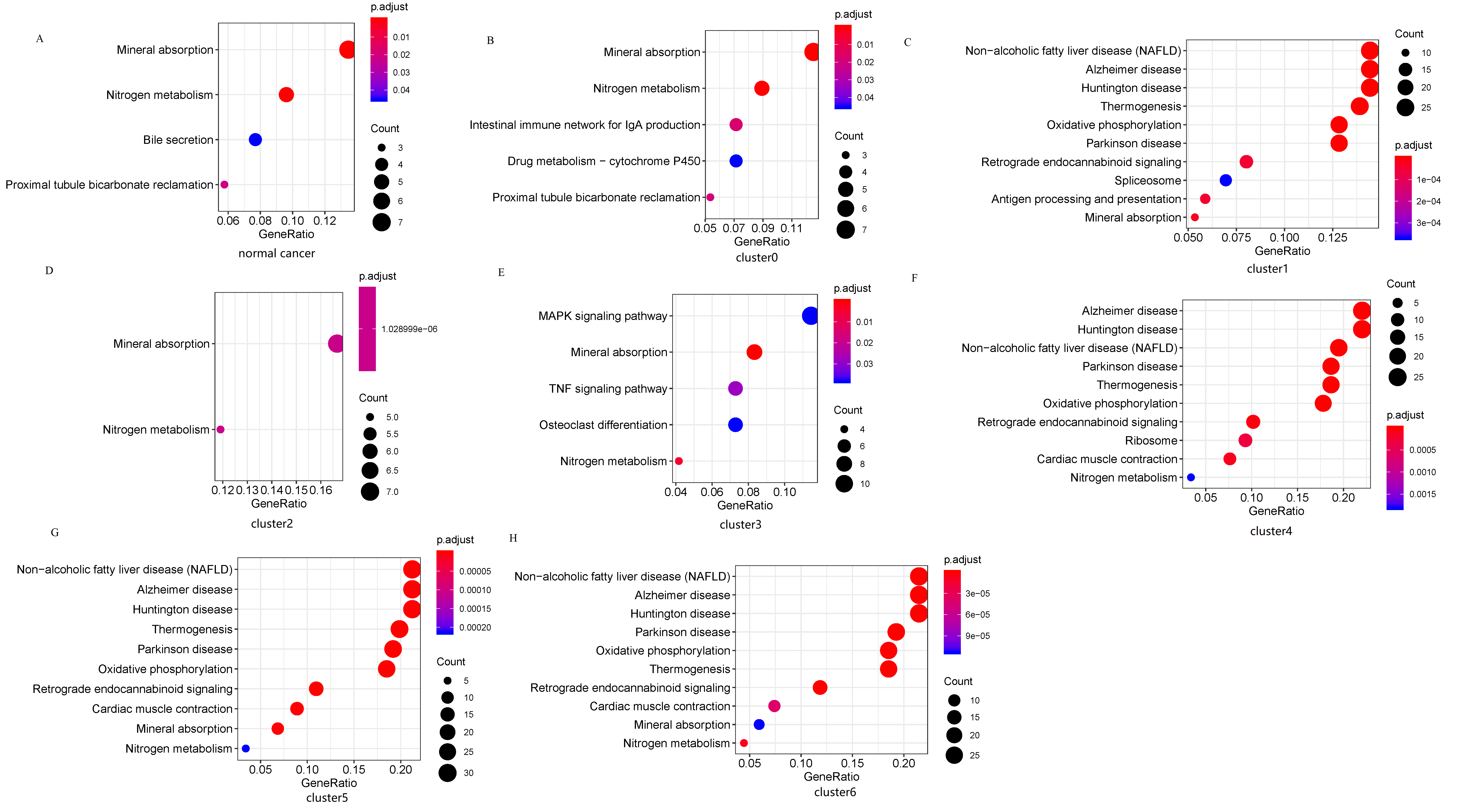

Supplement: Supplementary file 3 [file Image3.TIF]

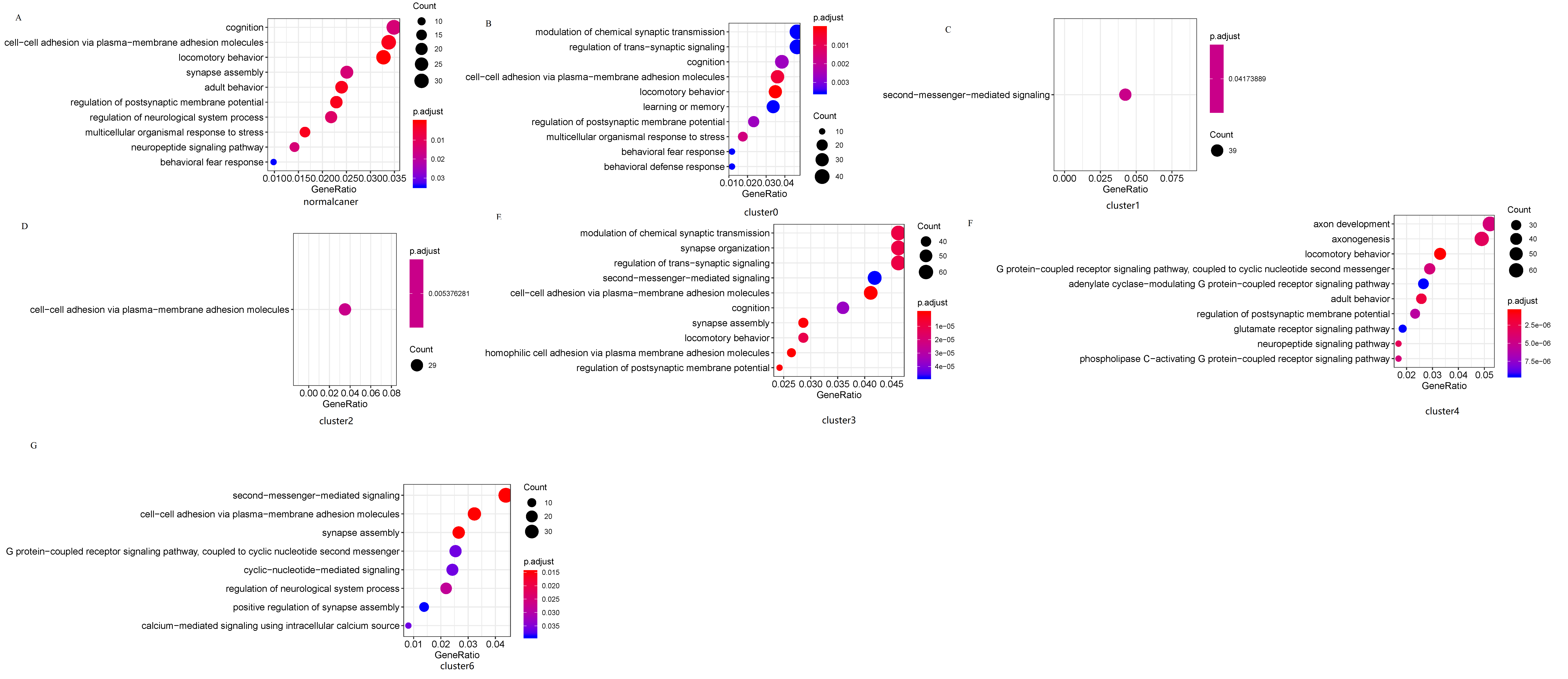

Supplement: Supplementary file 4 [file Image4.TIF]

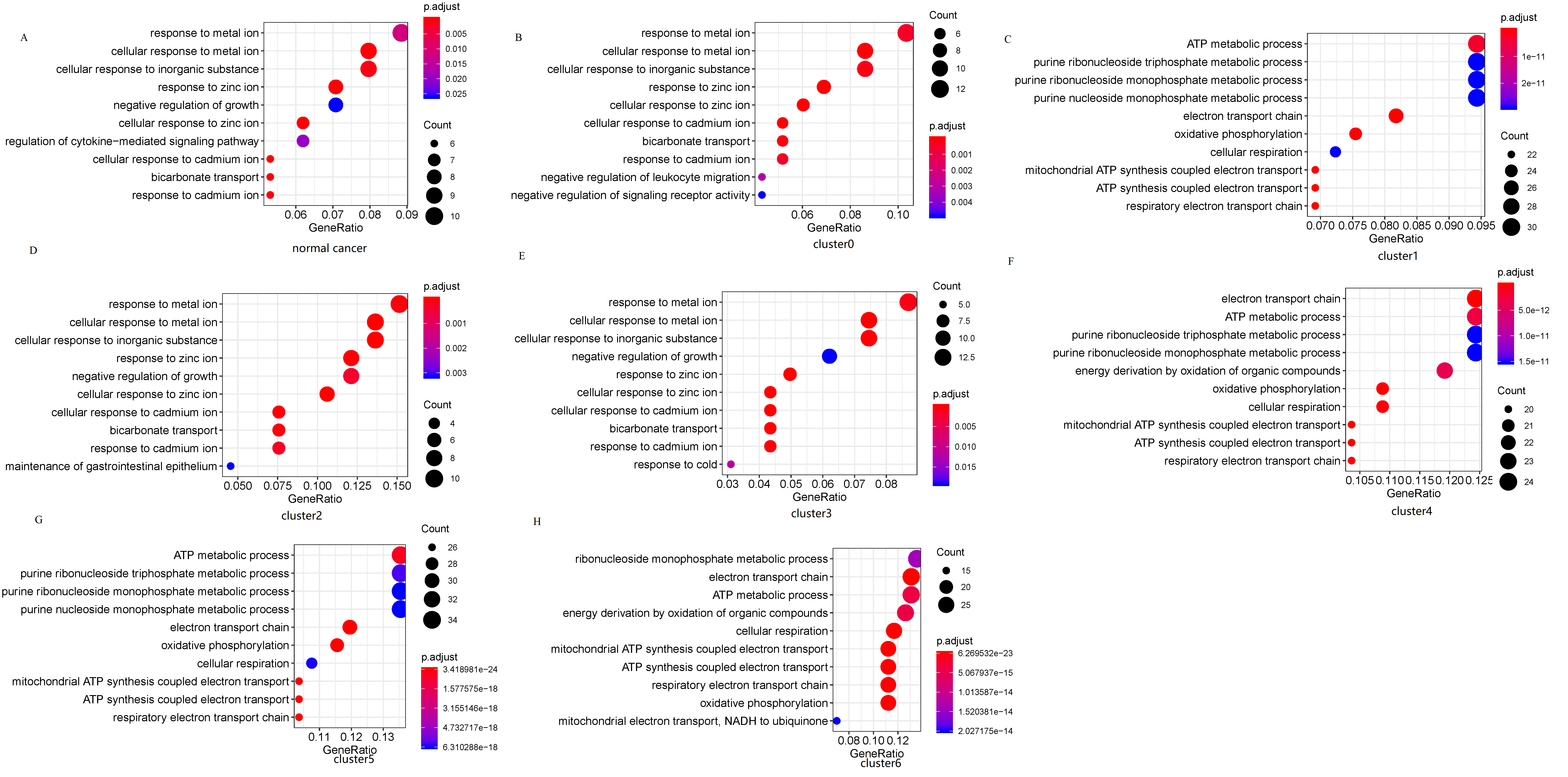

Supplement: Supplementary file 5 [file Image2.TIF]

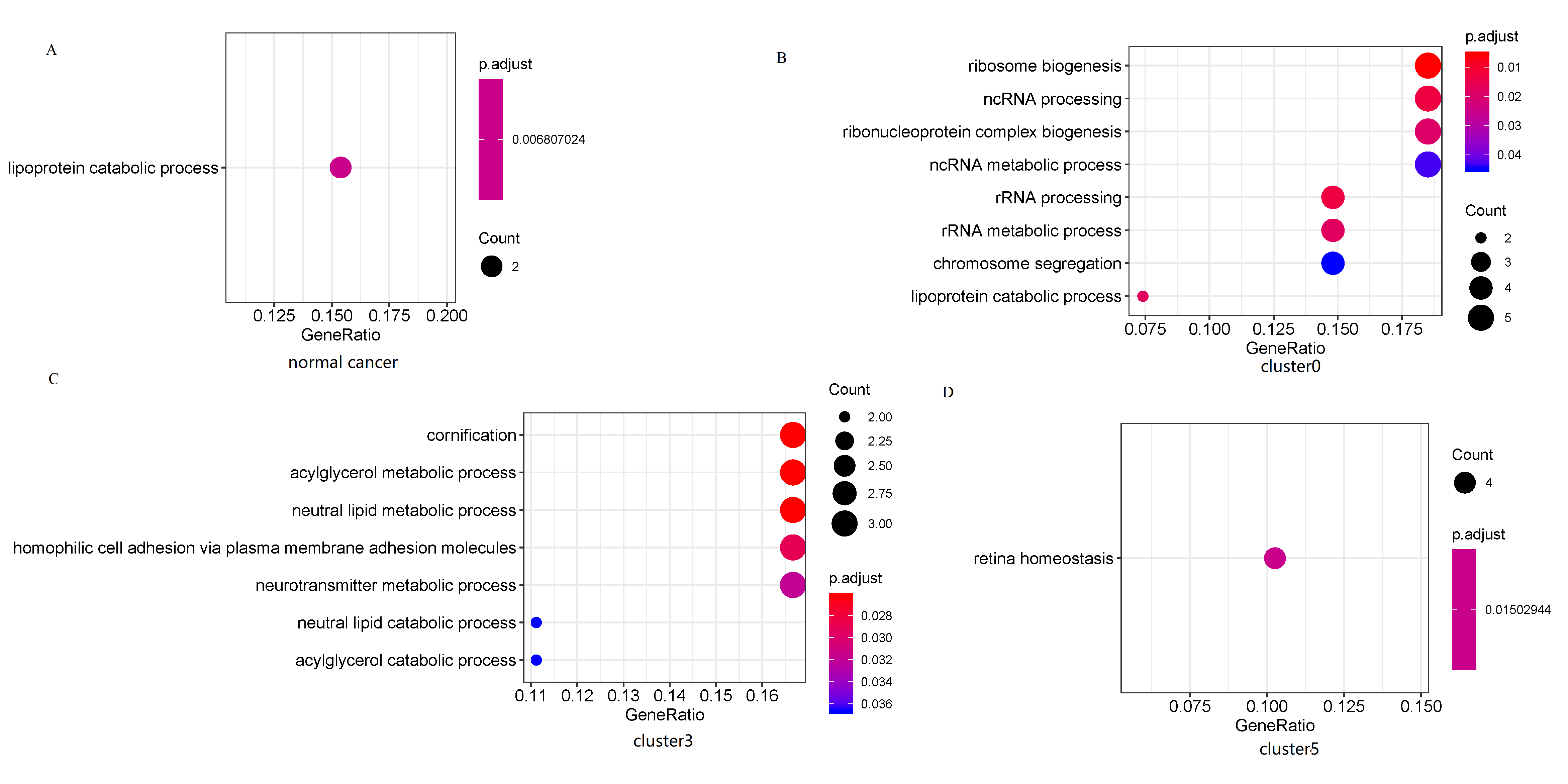

Supplement: Supplementary file 6 [file Image7.TIF]

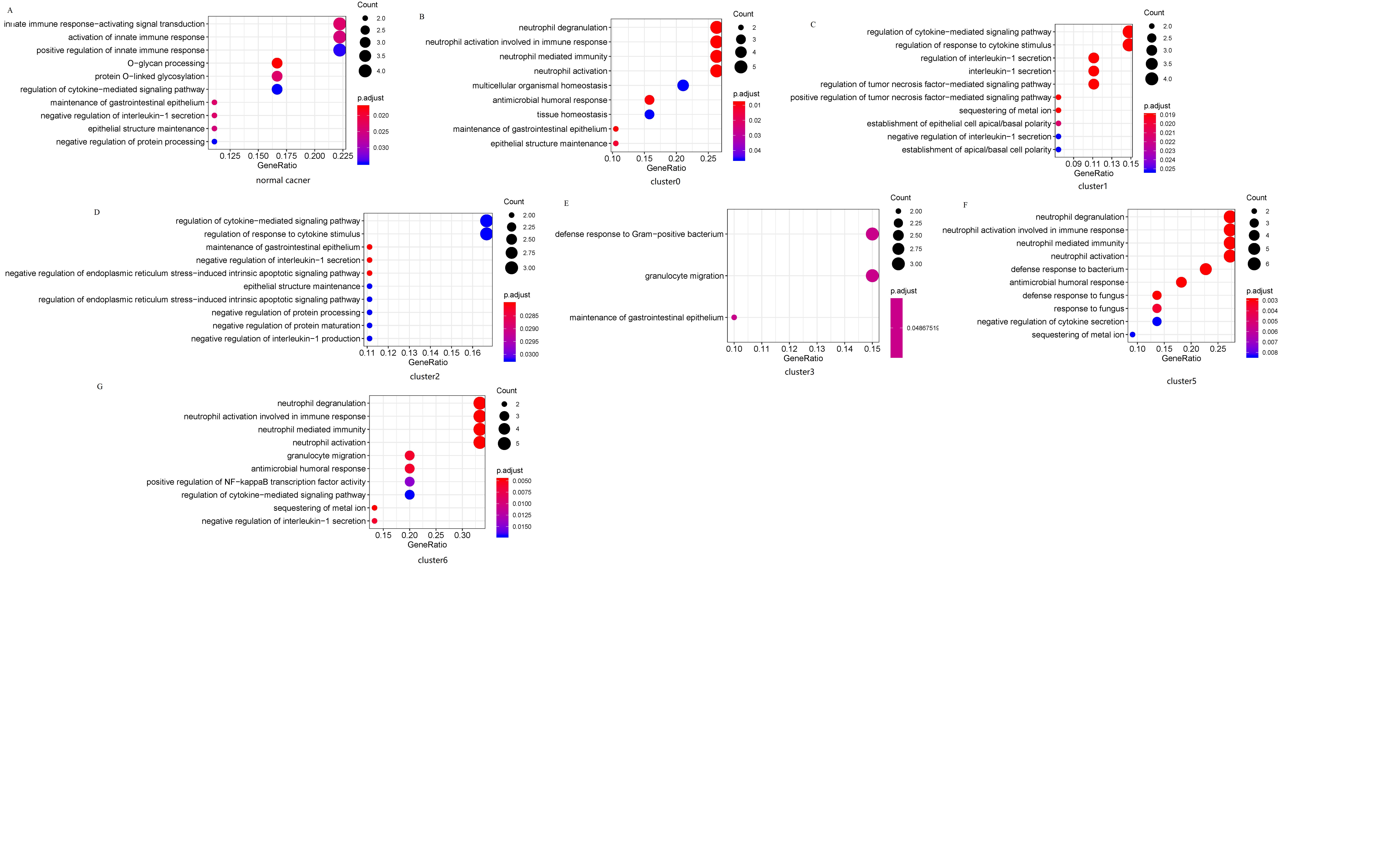

Supplement: Supplementary file 7 [file Image8.TIF]

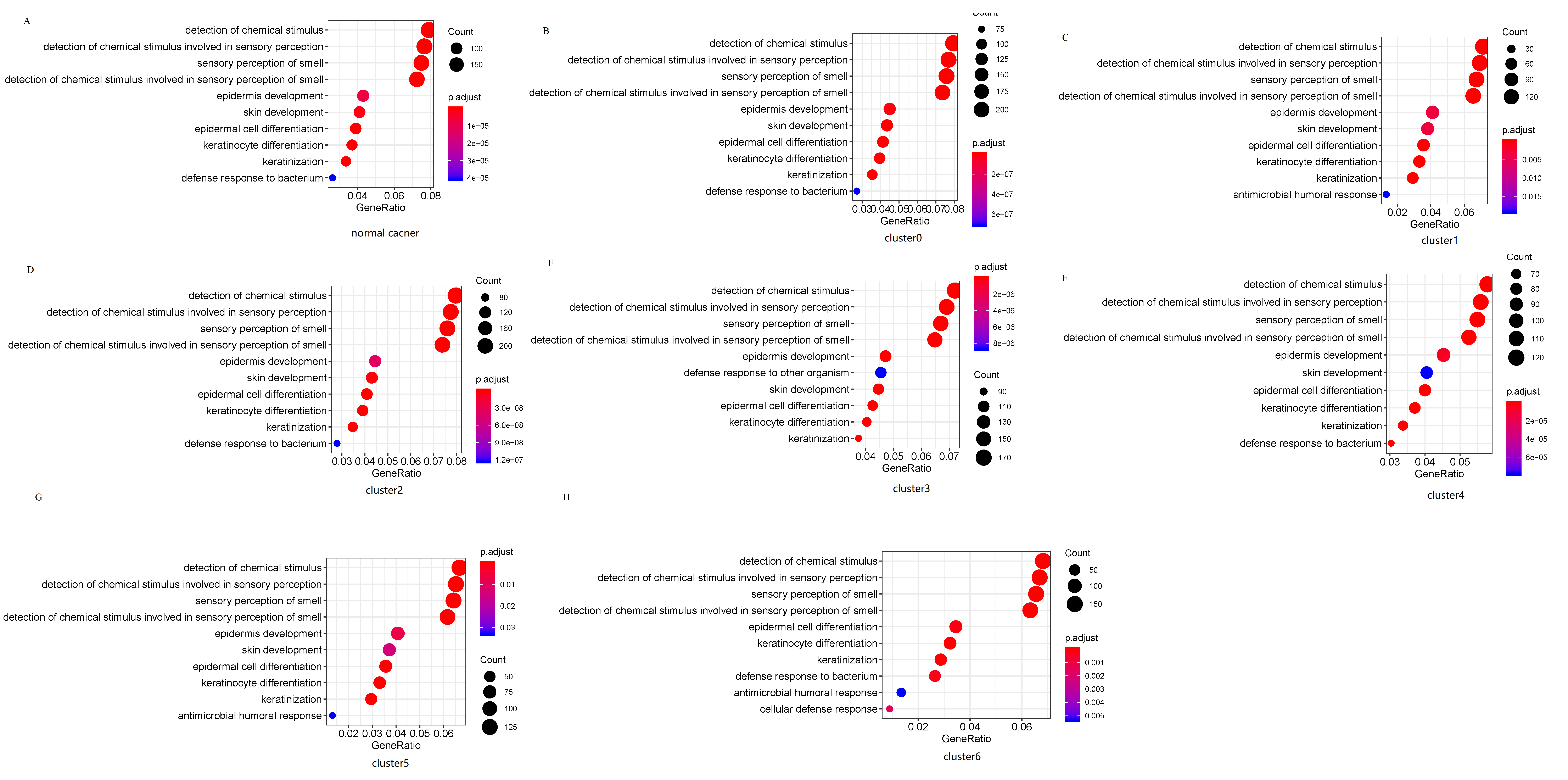

Supplement: Supplementary file 8 [file Image5.TIF]
